# Development and Characterization of Bioadhesive Gel of Microencapsulated Metronidazole for Vaginal Use

**Published:** 2010

**Authors:** Nayak Bhabani Shankar, Rout Prasant Kumar, Nayak Udaya Kumar, Bhowmik Benoy Brata

**Affiliations:** a *Department of Pharmaceutics, Faculty of Pharmacy, Jeypore College of Pharmacy, Rondapalli, Jeypore – 764002, Koraput, Orissa, India.*; b * Ajanta Pharma Ltd., Mumbai, India.*; c *Himalyan Pharmacy Institute, Majhitar, Rang po, East Sikkim, India*

**Keywords:** Microencapsulate-containing vaginal gel, bioadhesion, microcapsules, vaginal irritation

## Abstract

The present study concerned with the development and characterization of metronidazole microcapsules prepared by thermal change method using different ratios (1:1, 1:2 and 1:4) of ethyl cellulose in order to select the best microcapsule formulation with a good encapsulation efficiency and drug release profile. The obtained microcapsules were discrete, spherical with free flowing properties and evaluated for particle size, shape, flow properties, wall thickness, drug encapsulation efficiency and *in vitro *release performance. The drug carrier interactions were investigated in solid state by FT-IR spectroscopy and scanning electron microscopy. The microcapsules with a narrow size range of 23-68 μm showed higher encapsulation efficiency. The selected microcapsule formulation, MC3 (Drug polymer ratio 1:4) was employed for gel formulation with a variety of carbopol polymers (carbopol-934, 940, 974 and 980) by mechanical stirring method in order to develop a sustained release microencapsulated metronidazole microcapsules-containing bioadhesive gel. The prepared bioadhesive gels were evaluated for pH, spreadability, extrudability, viscosity, vaginal irritation, *in vitro *drug release, bioadhesion, accelerated stability and *in vitro *drug release kinetic. *In vitro *experiments indicated a sustained release over 24 h and an acceptable bioadhesion quality for formulation F3. Hence, it can be concluded that the formulation F3 has potential to deliver metronidazole in a controlled and constant manner for prolong period over other formulations and can be adopted for a successful delivery of metronidazole for vaginal use.

## Introduction

Over the last 20 years, extensive effort has been made towards the administration and absorption of drugs via vaginal route. The first truly controlled drug delivery systems for use in the vagina were developed in 1970, when the first vaginal rings was used for delivery of medroxy progesterone acetate for contraception. The presence of dense network of blood vessels and large surface area has made the vagina as an excellent route of drug delivery for systemic and local effects (1). The drug delivery from the vagina has advantages over oral delivery because of the ability to by-pass first pass metabolism, ease of administration and high permeability of low molecular weight drugs (2). However, conventional vaginal delivery systems such as creams, foams, pessaries and jellies reside for relatively short period at the targeted site because of the self cleaning action of the vaginal tract which limited effective drug levels for a shorter period leads to increased dose frequency of the drug. This ultimately results to patient inconvenience and toxic conditions (3). The use of controlled release dosage forms offers numerous benefits including reducing vaginal irritation, increasing stability of drug by decreasing drainage, prolonging the release of drug hence decreasing frequency of dosing etc (4). These systems using bioadhesive polymers such as polycarbophil, hydroxy propyl cellulose and polyacrylic acid discussed below, were propagated to solve problems like low retention to the vaginal epithelium, leakage, messiness etc. For example Replens^®^ bioadhesive polycarbophil gel used to retain moisture and lubricate vagina. These formulations remained in vagina for 2 to 3 days and maintained the health and acidic pH of vagina (5). The hydrophobicity of ethyl cellulose was the main criterion for its selection as the coat material for the preparation of microcapsules hence it will assist to sustain the stability of metronidazole microcapsules in hydrogels (6). Metronidazole was used as model drug due to its bacteriostatic and bactericidal activity against gram negative bacteria. An interesting rationale for taking metronidazole was that the low molecular weight of this drug in comparison to the other drugs administered through the vaginal route (5). With the above considerations, an attempt was made to design and evaluate a newer microcapsule-containing bioadhesive gel to treat vaginal infection and increase the patient convenience.

## Experimental

Metronidazole was received as a gift sample from Aristo Pharmaceutical Ltd., Kolkata. Ethyl cellulose (BDH; having an ethoxy content of 47.5% by weight and a viscosity of 22 cps in a 5% solution in a 80: 20 toluene: ethanol solution at 25°C) was purchased from S.D. Fine Chem., Mumbai. All grades of Carbopol were received as gift sample from Corel Pharma Chem., Ahmedabad. All other chemicals and reagents used were of analytical grade. 

*Preparation of vaginal microcapsules*

Metronidazole microcapsules were prepared by thermal change method using various drug/polymer ratios. Briefly weighed quantity of ethyl cellulose and cyclohexane (50 mL) were heated in water bath. The temperature was gradually raised to 70°C over 20 min under constant stirring (500 rpm). Metronidazole was dispersed slowly with maintaining temperature at 80°C for 30 min and it was cooled slowly under continuous stirring and temperature was dropped to 5°C in order to hardening ethyl cellulose coated microcapsules (7). Then, filtered, dried in a desiccator. 

*Morphological and topographical characterization*

Microcapsules were observed with optical microscope (OLYMPUS BX-50, Japan) and scanning electron microscope (LEO, 435 VP, U.K.). Their diameters were determined with a pre-calibrated graduated eyepiece. Particle size was calculated by using equation, 

X_g_ = 10 x [(n_i_ x log X_i_) / N]

Where, X_g_ is geometric mean diameter, n_i_ is number of particles in range, X_i _is the midpoint of range and N is the total number of particles (8, 9). All the experimental units were scrutinized in triplicate (n=3).

*Flow properties*

Flowability of microcapsules was investigated by determining angle of repose, bulk density, Carr’s index and Hausner ratio. The angle of repose was determined by fixed funnel method. The microcapsules were tapped using bulk density apparatus (Excel Enterprises, Kolkata) for 1000 taps in a cylinder and the change in volume was measured (10, 11, 12). Carr index and Hausner ratio were calculated by the formula: 

Carr index (%) = (D_f_ -D_0_) ×100 ⁄ D_f_

Hausner ratio = D_f_ ⁄ D_0 _

Where, D_f_ is poured density; D_0_ is tapped density. All the experimental units were studied in triplicate (n=3).

*Drug content and encapsulation efficiency (DEE)*

Accurately weighed microcapsules equivalent to 50 mg of metronidazole, were suspended in 100 mL of simulated vaginal fluid (SVF, phosphate buffer, pH 4.9) and kept for 24 h. Next day it was filtered after stirring and analyzed by using UV-Visible spectrophotometer (UV-1700, Shimadzu, Japan) after suitable dilution at 320 nm. Drug entrapment efficiency (DEE) was calculated using the formula (11, 12).

DEE = (Practical drug content/Theoretical percent drug content) × 100

Each sample was analyzed in triplicate (n=3).

*Wall thickness of microcapsules*

Wall thicknesses of the microcapsules were determined by the method suggested by Luu *et al., *using equation (13). 

h = r (1-P) d_1_ ⁄ 3[Pd_2_ + (1-P) d_1_] 

Where, h is wall thickness; r is mean radius of microcapsules from optical microscopic observations; d_1_ is density of the core material; d_2_ is density of the coat material; p is the proportion of medicament in the microcapsules. All the test samples were examined for three times.

*In vitro drug release studies of microcapsule formulations*

*In vitro *drug release study was carried out in USP XXI paddle type dissolution test apparatus using simulated vaginal fluid (SVF) as dissolution medium (900 mL phosphate buffer. pH 4.9, at 37±1°C, 100 rpm). An aliquot sample (5 mL) was withdrawn at an interval of 1 hr with replacement of fresh medium and analyzed for metronidazole content by UV-visible spectrophotometer at 320 nm (10, 11). All the experimental units were evaluated in triplicate (n=3). The same method was adopted for each batch of microcapsules.

*Infrared spectroscopy (IR) A*

Fourier Transformed Infrared Spectrophoto- meter (840, Shimadzu, Japan) was used to scan the drug samples prepared as KBr pellets, over the range of 4000-600 cm^-1^ (9). 

*Scanning Electron Microscopy (SEM)*

The SEM analysis was carried out using a scanning electron microscope (LEO, 435 VP, U.K.). Prior to examination, samples were mounted on an aluminium stub using a double sided adhesive tape and making it electrically conductive by coating with a thin layer of gold (approximately 20 nm) in vacuum (9). The scanning electron microscope was operated at an acceleration voltage of 05 KV. 

*Preparation of microcapsule-containing vaginal bioadhesive gels*

Various batches of metronidazole microcapsules incorporated gels were prepared by mechanical stirring method using various grades of carbopol such as carbopol 934, 940, 974 and 980 with other formulation additives. For all batches, the microcapsules were mixed with prepared bioadhesive gels (14, 15). The gel preparations were packed in wide mouth plastic jars covered with screw capped plastic lid after covering the mouth with an aluminum foil and were kept in cool place for further study. 

*Estimation of metronidazole in vaginal gels*

An accurately weighed gel (0.5 g) was suspended in 25 mL of SVF and kept for 24 h. Next day it was filtered after stirring and analyzed by using UV-visible spectrophotometer at 320 nm after suitable dilution (16, 17). 

*Drug content uniformity*

Initially, the formulations were tested for homogeneity by visual inspection. To ensure the homogeneity of drug content in the formulation of the gel, six tubes were sampled from the different locations of the mixer and assayed for the drug content as stated above (14, 17). Studies were performed in triplicate for all the formulations. 

*Determination of pH*

The pHs of the carbopol gels were determined by a digital pH meter (Model MK–VI, Kolkata). 1g of gel was dissolved in 25 mL of distilled water and the electrode was then dipped in to gel formulation and constant reading was noted (14, 18). The measurements of pH of each formulation were replicated three times. 

*Determination of spreadability*

Spreadability of the formulations was determined by an apparatus suggested by *Multimer et al. *(14, 19)*. *Each experiment was replicated for three times.

*Extrudability study*

In conducting the test, a closed collapsible tube containing above 20 grams of the gel was pressed firmly at the crimped end and a clamp was applied to prevent any rollback. The cap was removed and the microcapsule-containing gel was extrudes until the pressure was dissipated (15, 19).

*Viscosity measurement*

A Brookfield digital viscometer (Brookfield Engineering Laboratories, Model DV-II, Mumbai) with a suitable sample adaptor was used to measure the viscosities of the microcapsule-containing gel in cps (14, 20). 

*Vaginal irritation test*

Microcapsule-containing gels (0.5 g) were applied in to the vagina of the Newziland white rabbits. After 72 h, the microcapsule-containing gel was removed and the following characteristics were observed in test animals and in control by visual inspection that is sensitization (allergic reaction), photosensitization, edema and excess redness (14, 17). The study protocol was approved by the Institutional Animal Ethics Committee (Regd. No. HPI / 07 / 60 / IAEC / 0013).

*In vitro drug diffusion studies of microcapsule-containing vaginal gels*

*In vitro *drug release study was carried out in KC - Diffusion cell using SVF as the diffusion medium. The processed cellophane membrane was used tosimulate the vaginal *in vivo *condition like vaginal epithelial barrier. The drug content in withdrawn sample was determined by UV-visible spectrophotometer at 320 nm (15, 17, 18). All the experimental units were evaluated in triplicate (n = 3) .The same method was adopted for each batch of microcapsule-containing gels.

*Drug release Kinetic study*

In order to study the exact mechanism of drug release from the microcapsule-containing gels, drug release data were analyzed according to zero order, first order, Higuchi square root and Korsemeyer-Peppas equation (22, 21). 

*Bioadhesion measurements*

The bioadhesion measurement was performed by using a modified balance method with mucosal membrane of goat vagina as reported by Parodi *et al. *(23, 24).

*Accelerated stability studies of microcapsule-containing vaginal gel*

Stability studies were performed according to ICH guidelines. The formulations were stored in hot air oven at 37 ± 1°C, 45 ± 1°C and 60 ± 1°C for a period of 12 weeks. The samples were analyzed for drug content every two weeks by UV-visible spectrophotometer at 320 nm. Stability study was also carried out by measuring the change in pH of gel at regular interval of time (19, 25). 

*Statistical Analysis*

Statistical data analyses were performed using the ANOVA one way at 5% level of significance p < 0.05 (26). 

## Results and Discussion

**Figure 1:**
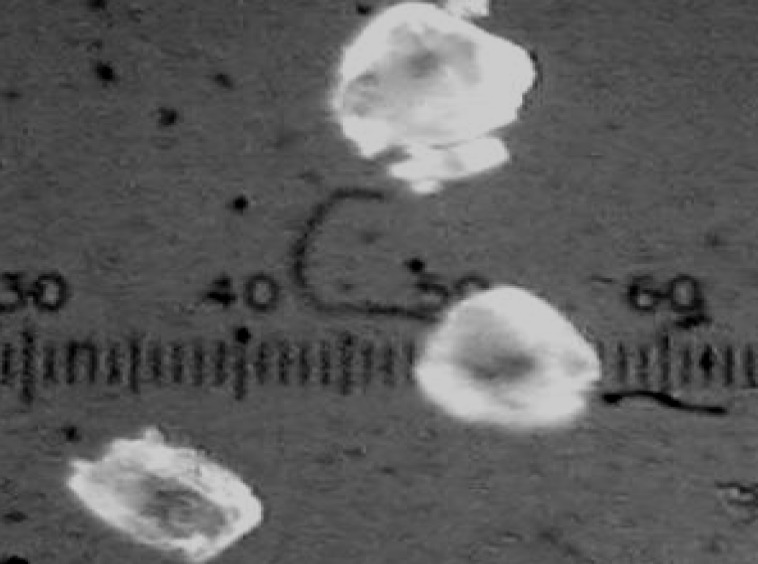
Optical micrographof metronidazole microcapsules (×100).

The obtained microcapsules were found to be non- aggregated. The formulation design of vaginal microcapsules has been presented in Table 1. The optical microscopy (Figure 1) revealed that all obtained microcapsules were opaque, discrete and spherical particles with smooth surfaces, which further confirmed by SEM studies. Particle size distribution of selected microcapsules and the mean particle size for all formulations were graphically represented in Figures 3 and 4. 

**Table 1:** Evaluation parameters of prepared metronidazole microcapsule

**Formulation code/ Evaluation parameters**				**MC1**		**MC2**		**MC3**	
Particle size (μm) (X±S.D) Drug content (mg) (X±S.D) Encapsulation efficiency (%) (X±S.D) Bulk density (g/cc) (X±S.D) Angle of repose (θ) (X±S.D) Hausner’s ratio (X±S.D) Carr’s index (X±S.D) Wall thickness (μm) (X±S.D) *In vitro *drug release (%) (12 h study) (X±S.D)				23.633±1.21 19.035±0.25 85.989±1.12 1.228±0.11 15.6±0.25 1.093±0.12 08.600±0.56 3.447±0.16 72.958±1.12		28.831±0.99 12.039±0.32 86.453±0.78 1.377±0.24 21.6±0.62 1.107±0.38 10.790±0.23 3.888±0.20 71.218±1.07		37.233±1.42 07.525±0.21 87.258±0.76 1.416±0.19 24.8±0.35 1.131±0.27 07.125±0.41 4.567±0.18 45.434±0.92	
ANOVA									
*Source of Variation*	*SS*	*df*	*MS*		*F*		*P-value*		*F crit*
Between Groups	24.0735	2	12.0367		0.0129		0.00987		3.40283
Within Groups	22388.7	24	932.863						
Total	22412.8	26							

All the values are expressed in mean ± Standard deviation (n=3).

Standard error mean < 1.07.

MC1 = drug: polymer ratio 1:1 w/w, MC2 = drug: polymer ratio 1:2 w/w and

MC3 = drug: polymer ratio 1:4 w/w.

**Figure 2:**
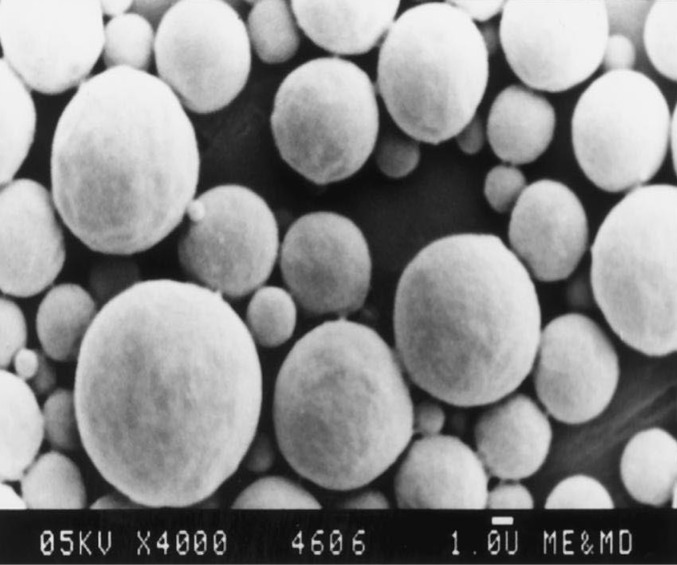
Scanning electron micrograph of metronidazole microcapsule at 05KV × 4000

**Figure 3:**
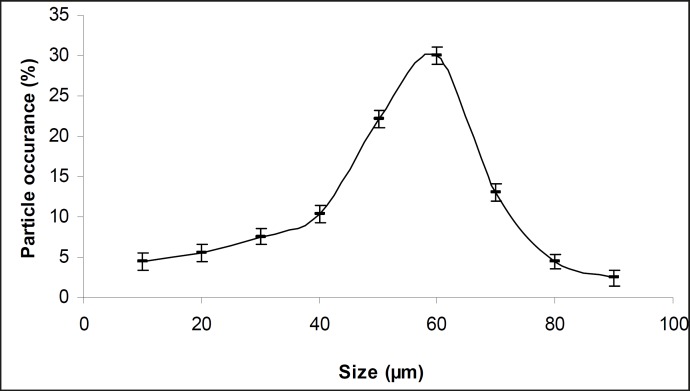
Particle size distribution curve of selected microcapsule formulations

The mean diameter of the microcapsules was found to be increased with increase in proportion of coat material as shown in Table 1. The particle size distribution was found as wide in formulation MC3. So care must be taken during preparation of microcapsules that is stirring must be done with much high speed, there must be gradual reduction of temperature during cooling and avoidance of sticking of microcapsules. The flow properties and wall thickness of the microcapsules were shown in Table 1. All the formulations had excellent flow properties. The highest wall thickness (4.567 μm) belonged to MC3. The wall thickness of the microcapsules mainly depended on polymer content. As usual, the wall thickness of the microcapsules increased with polymer ratio as depicted in Table 1. Relatively high drug content and encapsulation efficiency were observed for each formulation presented in Table 1. The increased encapsulation efficiency may be attributed to the hydrophobic nature of ethyl cellulose and metronidazole. It was foumnd that the encapsulation efficiency improved by decreasing the polymer content. The *in vitro *drug release profiles of various microcapsule formulations have been shown in Table 1 and Figure 5.

**Figure 4:**
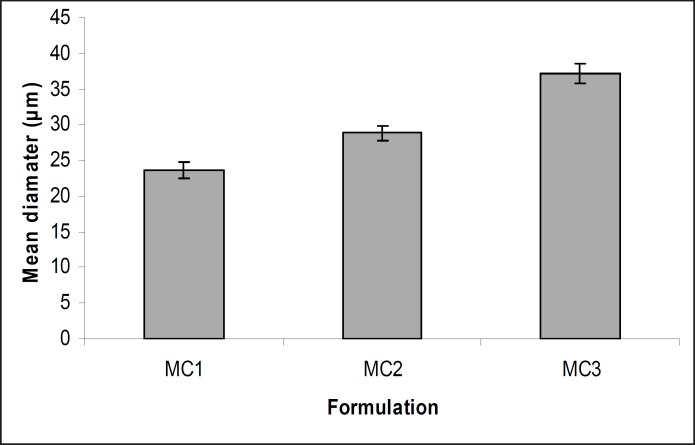
Mean diameter of different microcapsule formulations

**Figure 5:**
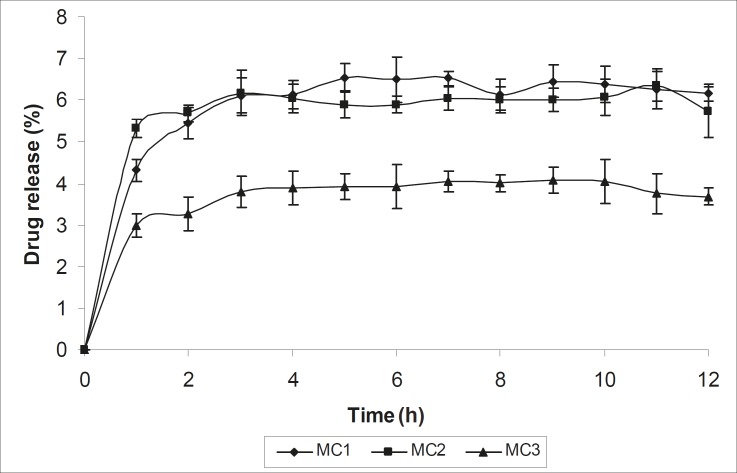
*In vitro *release profile of Metronidazole from different microcapsule formulations

**Figure 6:**
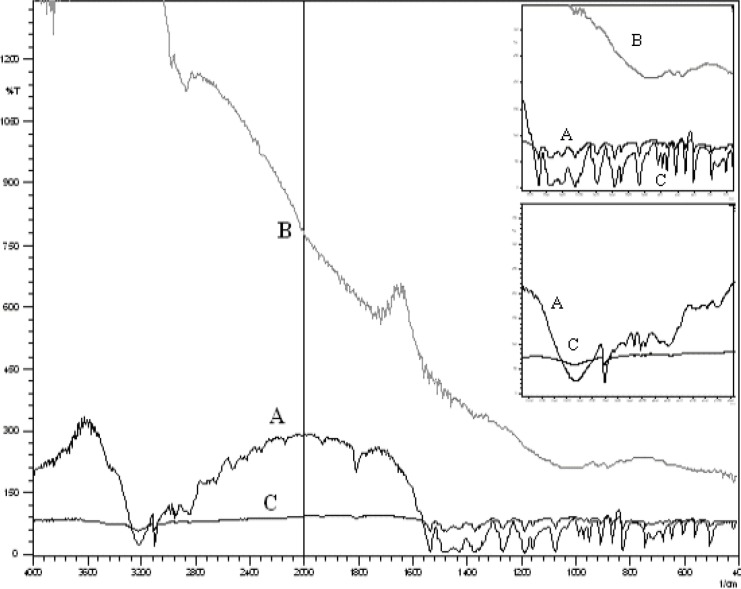
Entire FT-IR spectra and analysis region of pure drug (A), ethylcellulose (B), vaginal microcapsule formulation (C).

 In all cases, the decrease of polymer proportion resulted to the increase of release rate. The drug release from MC3 formulation was only about 45.333% even after 12 h. Thus, it was concluded that it possess a sustained release of drug for a longer period of time in comparison to theother formulations. The interaction between the drug and the carrier often leads to identifiable changes in the FT-IR profile of solid systems. FT-IR spectra at 45 scan and at a resolution of 1 cm^-1^ were recorded in KBr press pellets for pure drug (Figure. 6A), polymer (Ethyl cellulose) (Figure. 6B) and the selected (MC3) microcapsule formulation (Figure. 6C) of 1:4 drug / polymer ratios. In FT-IR studies, the characteristic C-N stretching at around 1620 cm^-1^ was clearly distinguishable in the selected formulation (MC3). Additionally characteristics O-H stretching vibration at around 3250 cm^-1^ and N=O symmetrical and asymmetrical stretching at around 1560 and 1350 cm^-1^ respectively (27, 28), were also observed unchanged in the formulation suggesting no drug polymer chemical interaction. The morphology of the ethyl cellulose metronidazole systems prepared by thermal change method was investigated by SEM analysis (Figure 2). Microcapsules appear as small spherical particle with smooth surfaces of homogenous morphology (9). 

Selected batches of prepared vaginal microcapsules were then incorporated in gels prepared by mechanical stirring with various grades of bioadhesive polymers, such as carbopol 934, 940, 974 and 980 and other formulation additives. The experimental design of the formulated gels has been shown in Table 2. 

**Table 2:** Experimental design of microcapsule-containing vaginal gels

**Formulation code **	**Microcapsule-containing gel compositions Amount taken in percentage (w/w) **					
	Micro-capsules	Carbopol	Triethanol-amine	Alcohol	Propylene glycol	Distilled water
F1	1	0.6	0.5	20	10	q.s.
F2	1	0.6	0.5	20	10	q.s.
F3	1	0.6	0.5	20	10	q.s.
F4	1	0.6	0.5	20	10	q.s.

F1: Gel with Carbopol 934, F2: Gel with Carbopol 940, F3: Gel with Carbopol 974

and F4: Gel with Carbopol 980. q.s. quantity sufficient.

The drug content of the prepared binary systems was in the range of 72.98 - 94.52% (Table 3). 

Formulation F3 showed maximum drug content. Drug content uniformity data (table 3), indicates the suitability of the applied method for the semi solid systems preparation. The pH of gels were within the range of 6.8 to 7.5 (Table 3), which reflects that the gel will be non irritant to vagina. This was further confirmed by vaginal irritation study in rabbit. The spreadability plays an important role in patient compliance and helps in uniform application of gel to the skin. A good gel takes less time to spread and will have high spreadability (19). The formulation F4 containing carbopol 980 showed maximum spreadabilty as abridged in Table 3. 

**Table 3:** Evaluation parameters of prepared metronidazole microcapsule-containing bioadhesive vaginal gels

**Formulation code**	**F1**		**F2**			**F3**		**F4**	
Drug content (%) (X±S.D)	78.72 ± 0.030		73.33 ± 0.055			94.52 ± 0.043		72.98 ± 0.029	
Drug content uniformity	**		**			***		**	
pH (X±S.D)	7.5 ± 0.011		7.3 ± 0.016			6.8 ± 0.027		7.2 ± 0.025	
Extrudability	*		**			***		***	
Spreadability (g.cm/sec) (X±S.D)	46.87 ± 0.098		75.02 ± 0.134			150.1 ± 0.324		187.5 ± 0.315	
Viscosity (cps) (X × 10^4^)	2.015		1.742			1.802		1.555	
Vaginal irritation test	-		-			-		-	
*In vitro *drug diffusion (%)(18 h study) (X±S.D)	46.946±1.12		72.958±0.989			55.234±1.09		76.907±1.05	
Bioadhesive strength (Kg) (X±S.D)	1.25 ± 0.56		3.50 ± 0.15			8.50 ± 0.46		4.65 ± 0.38	
Bioadhesion time (h)	8-9		18-19			>36		22-23	
Zero order (r)	0.562		0.751			0.581		0.676	
First order (r)	0.716		0.746			0.797		0.658	
Higuchi square root (r)	0.858		0.889			0.945		0.871	
Korsmeyer and Peppas (r)	0.883		0.848			0.925		0.818	
(n)	0.403		0.624			0.456		0.444	
ANOVA									
*Source of variation*	*SS*	*df*		*MS*	*F*		*P-value*		*F crit*
Between groups	1782.4644	3		594.15481	0.3064569		0.020553		2.838745
Within groups	77551.489	40		1938.78724					
Total	79333.954	43							

**n**- Diffusion exponent related to mechanism of drug release, according to equation, **m**_t_ **/ m**_¥_ **= kt**^n^.

All the values are expressed in mean ± Standard deviation (n=3). Standard error mean < 0.844.

F1: Carbopol 934, F2: Carbopol 940, F3: Carbopol 974 and F4: Carbopol 980.

* (good), ** (very good), *** (excellent) and - (no irritation).

**Figure 7:**
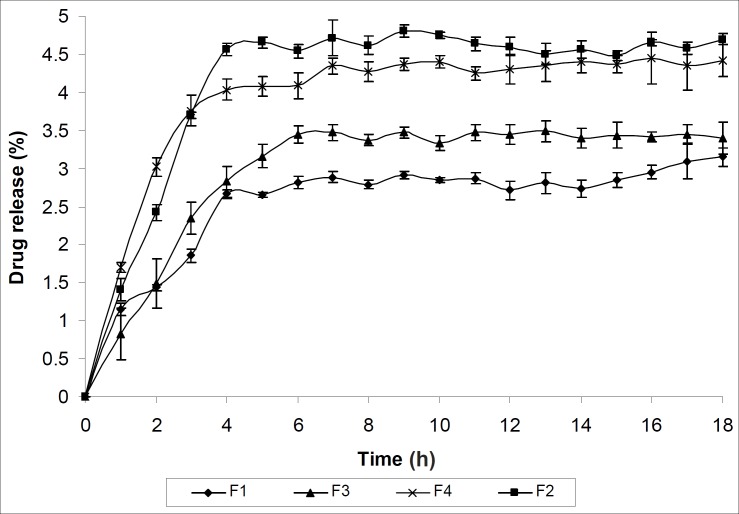
Drug diffusion profile of various microcapsule-containing gel formulations

The extrusion of gel from tube is important during application and for the patient compliance (19). Both the formulations F3 and F4 showed excellent extrudability property. Viscosity is an important parameter for characterizing the gels as it affects the spreadability, extrudability and release of drug (25). The formulation F1 is showing maximum viscosity where as the formulation F4 is showing minimum viscosity as evident from Table 3. This may be due to their three dimensional cross linking structure. The result of vaginal irritation study has been shown in Table 3. All formulations were found to be non irritant to vagina of New Zealand white rabbit. The release mechanism was not significantly influenced by formulation variables and was predominately diffusion- controlled. The release rate was inversely proportional to wall thickness. The *in vitro *drug release of all the formulations (F1- F4) was found sustained for each formulation and influenced by the polymer added. The *in vitro *drug release profile was presented in table 3 and Figure 7. 

To categorize the kinetics of drug release from microcapsule-containing gel, release data was verified with different kinetic models. Table 3 indicates that drug release from all formulations obeyed Higuchi kinetic equation except formulation F1 which obeyed Korsmeyer and Peppas order kinetics. The diffusion co-efficient data indicate that all the formulations release the drug by diffusion following Fickian transport mechanism except the formulation F2 which follow Non-Fickian transport mechanism. Statistical verification with one way ANOVA method attested the fact that the drug release data were found significant at 5% level of significance (p < 0.05). 

**Figure 8:**
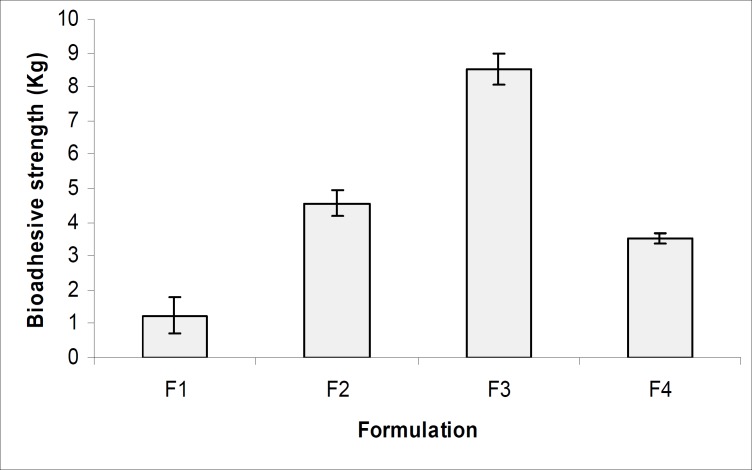
Bioadhesive measurement of various microcapsule-containing gel formulations

Figure 8 and Table 3, indicates the bioadhesive properties of the prepared gels (F1- F4) and the result showed that all formulations had good bioadhesive values. The formulation F3 containing carbopol 974 showed the highest bioadhesive property which may be due to greater polymeric cross linking structure and greater cohesive force between polymeric gel and vaginal epithelial membrane (23, 24). The formulation F3 also showed maximum bioadhesion time, which reflect that the microcapsule-containing gel can be retained in vagina for 3 to 4 days which will improve the patient compliance. The accelerated stability studies were performed according to ICH guidelines for 12 weeks and the results were found to be stable in varying temperature as shown in Table 4. All results were found to be statistically significant applying one way ANOVA at 5% level of significance (p < 0.05). 

**Table 4:** Accelerated stability study of selected vaginal gel formulation

**Storage Temp.(°C) **	**Potency of formulation (%) **							
	**Period of studies in week **							
	**1** ^st ^ **day **	**2** ^nd ^	**4** ^th ^	**6** ^th ^	**8** ^th^		**10** ^th^	**12** ^th ^
37 ± 1	99.56	99.31	99.12	99.05	98.87	98.51		98.39
45 ± 1	99.56	99.17	98.94	98.76	98.61	98.29		98.15
60 ± 1	99.56	99.08	98.84	98.54	98.33	98.13		97.98
pH	6.8	6.9	6.7	6.6	6.7	6.8		6.9

## Conclusion

In conclusion, MC3, containing drug: polymer ratio 1:4, was selected as the best microcapsule formulationbecause of its slower release rate, higher entrapment efficiency and excellent flow property. F3 gel (containing 1% w/w of drug loaded microcapsules and 0.6% w/w of carbopol 974) was found to be the best because releasing almost 100% of metronidazole over a period of 36 h in simulated vaginal fluid, successfully. The novel formulation design facilitated the optimization and successful development of microcapsule-containing bioadhesive vaginal gel (MBVG) formulations for enhanced vaginal drug delivery by optimum vaginal bioadhesion and longer retention. It was concluded that the applied protocol can be an effective strategy for the development of safe, easy, reproducible and cost effective vaginal delivery systems. 
